# Combination of Isoflurane and Propofol as General Anesthesia During Orthopedic Surgery of Perioperative Cerebral Hypoperfusion Rats to Avoid Cognitive Impairment

**DOI:** 10.3389/fmed.2020.549081

**Published:** 2020-10-20

**Authors:** Xinyue Bu, Tang Li, Haiyun Wang, Zhengyuan Xia, Di Guo, Jinxin Wang, Yi Sun, Chenyi Yang, Guoqiang Liu, Ji Ma, Zhuo Yang, Guolin Wang

**Affiliations:** ^1^Department of Anesthesiology, The Third Central Clinical College of Tianjin Medical University, Tianjin, China; ^2^Tianjin Key Laboratory of Extracorporeal Life Support for Critical Diseases, Artificial Cell Engineering Technology Research Center, Tianjin Institute of Hepatobiliary Disease, Tianjin, China; ^3^The Third Central Hospital of Tianjin, Tianjin, China; ^4^Tianjin Third Central Hospital, Nankai University, Tianjin, China; ^5^Department of Anesthesiology, University of Hong Kong, Hong Kong, China; ^6^State Key Laboratory of Medicinal Chemical Biology, Key Laboratory of Bioactive Materials for Ministry of Education, College of Medicine, Nankai University, Tianjin, China; ^7^Tianjin Research Institute of Anesthesiology, Tianjin, China

**Keywords:** cerebral hypoperfusion, cognitive function, isoflurane, propofol, GABA_A_R α1, BiP

## Abstract

**Background:** Perioperative cerebral hypoperfusion (CH) is common, although the underlying mechanism of cognitive impairment that results due to perioperative cerebral hypoperfusion remains to be determined. Isoflurane anesthesia induces neuronal injury via endoplasmic reticulum (ER) stress, whereas a sub-anesthetic dose of propofol improves postoperative cognitive function. However, the effects of the combination of isoflurane plus propofol, which is a common aesthetic combination administered to patients, on ER stress and cognition remain unknown.

**Methods:** We sought to determine the effects of isoflurane plus propofol on ER stress and cognitive function in rats insulted by cerebral hypoperfusion. Ligation of the bilateral common carotid arteries (CCA) was adopted to develop the cerebral hypoperfusion rat model. A second surgery, open reduction and internal fixation (ORIF), requiring general anesthesia, was performed 30 days later so that the effects of anesthetics on the cognitive function of CH rats could be assessed. Rats received isoflurane alone (1.9%), propofol alone (40 mg·kg^−1^·h^−1^) or a combination of isoflurane and propofol (1% and 20 mg·kg^−1^·h^−1^ or 1.4% and 10 mg·kg^−1^·h^−1^). Behavioral studies (contextual fear conditioning [FC] test), histological analyses (Nissl staining) and biochemical analyses (western blotting of the harvested rat brain tissues) were employed.

**Results:** Hippocampus-dependent memory of rats in group IP_1_ (1% isoflurane plus 20 mg·kg^−1^·h^−1^ propofol) was not impaired, and expression level of γ-aminobutyric acid A type receptor α1 subunit, a key cognition-related protein, remained normal. ER stress alleviator, binding immunoglobulin protein, increased extremely while ER stress transcription factor, C/EBP homologous protein, showed no statistical difference compared with the control group. Numbers of surviving neurons confirmed the substantial neuronal damage caused by propofol or isoflurane alone.

**Conclusions:** These data suggest that ER stress contributes to the underlying mechanism of cognitive impairment and that the combination of isoflurane and propofol did not aggravate cognitive impairment and ER stress in aging rats with CH that were further subjected to ORIF surgery.

## Introduction

Perioperative neurocognitive disorders (PND) have become the most common complications after routine surgical procedures, particularly in the elderly (1, 2). Following surgery (e.g., common orthopedic procedures), up to 50% of patients experience cognitive disturbances that can lead to serious complications, including poorer prognosis and a higher 1-year mortality rate in subjects with pre-existing neurodegeneration (3). Carotid artery stenosis (CAS) can be detected in 75% of men and 62% of women aged ≥65, with a stenosis extent of ≥50% occurring in 7% of men and 5% of women in this age group (4). CAS is an independent risk factor for chronic cerebral hypoperfusion (CH) (5), which reduces tissue oxygen levels and leads to oxidative stress and endothelial injury (6). In rodents, experimental chronic CH can be initiated by occlusion of the major arterial supply. And chronic CH could lead to mitochondrial dysfunction and protein synthesis inhibition. These effects may destroy the balance of anti-oxidases and reactive oxygen species (ROS) and produce oxidative damage. Oxidative injury to vascular endothelial cells, glia, and neurons also impair vascular function and neurovascular coupling, which may result in a vicious cycle that further reduces cerebral perfusion (7). Taking all these factors into account, aging orthopedic patients with preoperative carotid stenosis make up a population that needs to be treated carefully. Special caution on the selection of anesthetic drugs is needed to protect cognitive function.

We and others (8–10) previously reported that two commonly used anesthetics, isoflurane, and propofol, have opposite effects on cognitive function at certain doses. Isoflurane induces neuronal injury upon prolonged exposure to high doses (11), with an underlying mechanism linked to endoplasmic reticulum (ER) stress. By contrast, propofol at a sub-anesthetic dosage protects against neuronal damage due to cerebral ischaemia reperfusion injury, and such protective effects were not observed at a higher dose (12). We, therefore, tested the effect of partially replacing isoflurane with a sub-anesthetic dose of propofol (combined use of isoflurane and propofol) on the cognitive function of rats with CH in the current study. Previous studies showed that isoflurane minimum alveolar concentration (MAC) value was 1.45 ± 0.17%. 1.9% isoflurane, equivalent to 1.3 MAC, was sufficient to induce general anesthesia in rats (13), while a minimal infusion rate at 40 mg·kg^−1^·h^−1^ was required using propofol alone to induce general anesthesia in rats (14). Therefore, in our study, doses were carefully selected combining isoflurane and propofol (1% and 20 mg·kg^−1^·h^−1^ or 1.4% and 10 mg·kg^−1^·h^−1^) to ensure the required depth of general anesthesia.

γ-aminobutyric acid (GABA) is the main inhibitory neurotransmitter (15). The major subtype of GABA_A_ receptor (GABA_A_R) contains the α1 subunit. According to a previous study, GABA receptors in the central nervous system are divided into three types: A, B, and C. Among the three receptors, GABA_A_R is the earliest expressed and most widely distributed, found mainly in the hippocampus, the prefrontal cortex and the striatum. Studies have confirmed that most GABAergic synaptic transmission in the mammalian brain is mediated by GABA_A_R. It is also an important target receptor for central nervous system (CNS) general anesthetics, such as propofol and isoflurane. GABA_A_R consists of five subunits embedded in the cell membrane of neurons. At the center, a 0.5 mm diameter GABA-gated Cl^−^ channel is formed. When GABA binds to GABA_A_R, the Cl^−^ channel of the postsynaptic membrane is opened, and Cl^−^ enters the cell due to the concentration gradient. The potential increases to produce hyperpolarization, which in turn causes neuronal inhibition (16, 17). In 2014, Labrakakis et al. confirmed that the post-synaptic membrane GABA_A_R subunit composition determines the heterogeneity of inhibitory postsynaptic potential (IPSP), namely, GABA_A_R function (18). The native GABA_A_Rs present in the mammalian brain are mainly composed of α, β, and γ subunits. The most common configuration is a transmembrane pentamer composed of 2α_1_2β_2_γ_2_, accounting for 43% of all GABA_A_R configurations and representing the most abundant configuration in the hippocampus and the cerebral cortex (18). The GABA_A_R α1 subunit, which is related to cognition, is the most widely distributed in the mammalian brain, (19, 20). Its main function is to maintain CNS arousal and the sensitivity of the receptor to sedative hypnotics (propofol, isoflurane, etc.). Mutation of the M2 domain Ser270 and the M3 domain Ala291 in the α1 subunit affects the potency of isoflurane and propofol on GABA_A_Rs (21). Kelley et al. confirmed that cerebral ischaemia can induce miniature inhibitory postsynaptic current (mIPSC) reduction and GABA-activated current inhibition (22). Further studies found that mIPSC frequency and kinetic parameters did not change, only amplitude decreased, while oxygen-glucose deprivation (OGD) inhibited neuronal GABA_A_R α1 subunit expression (22). This finding suggests that the change in GABA_A_R activity is triggered by a decrease in the expression of its functional subunit α1. Furthermore, our previous study showed that a sub-anesthetic dose (20 mg·kg^−1^·h^−1^) of propofol exerts post-treatment brain protection by activating the KCC2-GABA_A_R pathway. Propofol post-treatment can reverse the decrease in hippocampal IPSCs after OGD injury, promote KCC2 expression, and maintain the normal function of GABA_A_R. However, administration of KCC2 antagonists only partially reversed the effect of propofol on mIPSC (23). IT remains unknown whether or not cerebral ischaemia triggers the expression change and structural regulation of GABA_A_R functional subunit protein. Is there any upstream mechanism other than KCC2 that regulates the GABA_A_R structure, thereby affecting its function? To answer these questions, we chose the GABA_A_R α1 subunit as a target of research in this study.

GABA_A_R undergoes post-synthesis modification and folding in the ER. Prolonged ER stress has been well-known to be related with neurodegenerative diseases (24, 25). The unfolded protein response (UPR) triggered by ER stress is an important quality control system for maintaining protein homeostasis (Proteostasis). Proteostasis refers to an equilibrium state of specific protein synthesis, folding and unfolding, modification and degradation in the intracellular proteome at a specific time point. The ER of the cell is a site for the folding and post-translational processing of secreted proteins and membrane proteins (~1/3 of the human proteome). Binding immunoglobulin protein (BiP), also known as glucose-regulated protein 78 (GRP78), is an ER chaperone protein whose expression is part of the UPR and is required to alleviate ER stress (26). Once ER stress occurs, BiP binds to unfolded proteins and activates downstream receptor proteins, increasing molecular chaperone expression, reducing global protein translation, and increasing unfolded/misfolded proteins. It degrades and reduces ER stress and protects cells through endoplasmic reticulum-associated degradation (ERAD).

The expression of C/EBP homologous protein (CHOP) is acknowledged as a specific and transcription factor of ER stress (27). It expresses at a very low level in normal physiology, but cellular stress leads to high-level expression (28). During stress, UPR attempts to increase protein-folding capacity and remove misfolded and unfolded proteins. If homeostasis is inadequately restored under chronic ER stress, terminal UPR will trigger apoptosis through abundant signaling mechanisms, mainly mediated by CHOP, c-Jun N-terminal kinase (JNK), and caspase-12, with CHOP as the most widely studied (29).

Thus, the expression levels of BiP, CHOP and the GABA_A_R α1 subunit were used to evaluate the cellular mechanisms accounting for the neural substrate conditions that allow normal cognitive functions in this study.

The objective of the current study was to explore general anesthetics for rats with CH that are subjected to ORIF surgery to protect cognitive function. By using behavioral and biochemical analyses, we tested the hypothesis that a combination of isoflurane and propofol better protects cognitive function than isoflurane or propofol administered alone during ORIF surgery.

## Materials and Methods

In our study, a ligation of bilateral CCA surgery (30) was adopted to prepare rats as CH animal model (31). A second surgery, ORIF (32), requiring general anesthesia, was operated 30 days later so that the effects of anesthetics on cognitive function of these CH rats could be assessed.

### Animals

Male Wistar rats, 16–18 months of age and 450–570 g in weight, were purchased from the Academy of Military Medical Science of the Chinese People's Liberation Army and housed in groups of six per cage (545 mm in length, 395 mm in width, and 200 mm in hight) with *ad libitum* access to food and water. The housing environment was maintained at a temperature of 20–22°C and a humidity of 45–65% under a 12 h light/dark cycle. All animal experiments were carried out according to the Guide for the Care and Use of Laboratory Animals (33) and were approved by the Institutional Animal Care and Use Committee of Tianjin Medical University. Rats were housed individually per cage (380 mm in length, 325 mm in width, and 180 mm in hight) 3 days before ligation of the CCA and fasted 12 h before surgery a normally supply of drinking water. After surgery, rats were also housed individually per cage for recovery.

### Ligation of the CCA

Rats were first anesthetized with intraperitoneal (i.p.) injection of 10% thiobutabarbital (100 ml/kg). After disappearance of body motion and the righting reflex, the rat was fixed on the operation platform. The surgical field was maintained sterile throughout the entire procedure. The skin of the rat's neck was shaved and disinfected with iodine tincture. A median incision of ~2–3 cm was made in the neck. The muscles and surrounding tissues were separated to expose the CCA. The CCA and a blunt end syringe needle (0.45 mm in diameter, 1 cm in length) were ligated tightly at the proximal side 1.5 cm from the bifurcation of the internal and external carotid arteries. The slipknot was firmly fixed, and the needle was carefully removed. The wound was sutured and disinfected. During surgery, a heating lamp was used to help maintain the body temperature of anesthetized rats at 37 ± 0.5°C (30).

### Anesthesia and ORIF Surgery

During ORIF surgery, rats were administered isoflurane via inhalation or propofol through tail vein injection. For the induction phase of anesthesia, the rat was placed in a transparent chamber (W 25 cm × D 15 cm × H 10 cm) connected to a vaporizer and anesthetized with 5% isoflurane and 40% oxygen. When the rat's righting reflex disappeared, the chamber was replaced by a mask. Each rat was then assigned to one of the following 5 groups (*n* = 32/group) and administered the respective anesthesia as maintenance: (1) Group C: local administration of anesthesia with 2% lidocaine and inhalation with air containing 40% oxygen via the mask for 3 h; (2) Group I: inhalation with air containing 40% oxygen and 1.9% isoflurane for 3 h; (3) Group P: venous transfusion with 40 mg·kg^−1^·h^−1^ propofol and inhalation with air containing 40% oxygen via the mask for 3 h; (4) Group IP_1_: venous transfusion with 20 mg·kg^−1^·h^−1^ propofol and inhalation with air containing 40% oxygen and 1% isoflurane for 3 h; and (5) Group IP_2_: venous transfusion with 10 mg·kg^−1^·h^−1^ propofol and inhalation with air containing 40% oxygen and 1.4% isoflurane for 3 h. The concentration of isoflurane was detected continuously by a gas monitor (Puritan-Bennett; Tewksbury, MA, USA) during the surgery.

ORIF surgical model: Under different modes of general anesthesia, the rats underwent an open tibial fracture of the left hind paw with intramedullary fixation. Supplemental analgesia was provided using <1 ml buprenorphine (0.3 mg/kg in saline) administered intraperitoneally (32). Surgery was carried out via aseptic techniques. The left hind paw of the rat was shaved and disinfected with iodine tincture. After the skin was incised, a 0.38 mm pin was inserted into the intramedullary canal. Once the tibia was internally fixated, the bone was fractured at the middiaphysis (tibial, midshaft) using surgical pliers. The skin was sutured with 8/0 Prolene sutures. In Group C, only the skin was incised and sutured. During surgery, a heating lamp was used to help maintain the body temperature of the anesthetized rats at 37 ± 0.5°C. Postintervention rats were moved to heated pads for recovery and then returned to their home cage supplied with sufficient food and water. For post-procedural pain relief, the rats were administered buprenorphine (0.05 mg/kg, subcutaneous) twice daily for 3 days (34).

### Contextual Fear Conditioning Test

The contextual FC test was utilized to evaluate cognitive function (35). The contextual FC test consisted of a training phase at 24 h prior to ORIF surgery and an evaluation phase on days 1 and 7 after ORIF (36), when hippocampal-dependent memory was assessed (37).

During the training phase, rats were placed in a chamber (Ugo Basile, Italy) and allowed to adapt to the environment for 120 s. After adaption, a 20 s 70-dB tone (conditional stimulus) was delivered, followed by an interval of 25 s. After the interval, an 0.70 mA electrical foot shock was delivered to the rat for 2 s (unconditional stimulus). After six pairs of conditional-unconditional stimuli, the rats learned the association and had established long-term memory. The pairs of conditional-unconditional stimuli were separated by 60 s inter-training intervals. Each training chamber was cleaned with 75% ethyl alcohol before placement of the next rat and was illuminated only with a 10 W bulb in a dark experimental room.

During the evaluation phase, rats were placed again in the training chamber for 5 min without tone and foot shock (38). Each animal's freezing behavior (without any movements) was analyzed by using the ANY-Maze Video Tracking System (Stoelting, Illinois, USA). The percentage of time spent exhibiting freezing behavior was calculated using the formula of 100^*^f/5 min, where f was the total of freezing time within 5 min. The results in this experiment were used to assess hippocampus-dependent memory (35, 37).

### Nissl Staining

On days 1 and 7 after ORIF, rats (*n* = 8/group) were first anesthetized with 10% thiobutabarbital (100 ml/kg, i.p.). Rats were perfused with saline before the heart stopped, followed by perfusion with 4% paraformaldehyde solution. Then, the brain was taken out and fixed in 4% paraformaldehyde for 24 h. Coronal slices (3.0-mm thick) from each brain containing the dorsal hippocampus and the medial dorsal prefrontal cortex were dehydrated and embedded in paraffin. A series of 10-μm-thick coronal sections was obtained from each slice, and the sections were stained with cresyl violet (39). For each brain, five sections at the dorsal hippocampus located at coordinates −3.14 from the bregma to −4.52 from bregma were analyzed for Ammon's horn pyramidal cell counts (40). Sections were examined by an observer who was blinded to the experimental conditions under light microscopy at a magnification of 200x. The number of surviving neurons in a 30,000 μm^2^ area of the CA1 was counted in each section. Only pyramidal neurons showing normal morphology with distinct cytoplasmic and nuclear outlines and a visible nucleolus were counted. Analysis of the data was performed by using Image Pro Plus 6.0 software (Media Cybernetics Co., USA).

### Western Blotting

On days 1 and 7 after ORIF, rats (*n* = 8/group) were sacrificed with sodium pentobarbital (240 mg/ml, Department of Pharmacy, Tianjin Medical University General Hospital, i.p., 800 mg/kg) (41). After ensuring that the heart of the rat had stopped, the brain was removed, and the hippocampal tissue was separated. To obtain total cellular protein, the hippocampus was homogenized in RIPA solution (Biomart, Beijing, China) buffer and then centrifuged at 4°C at 12,000 r/min for 10 min (Sigma 3–30KS, Sigma Laboratory Centrifuges, Germany). Membrane protein fractions were obtained with a Mem-PER Plus Membrane Protein Extraction Kit (Thermo Fisher Scientific, USA). The quantity of protein in the supernatant was determined using a bicinchoninic acid (BCA) protein assay kit (Beyotime Biotechnology, Beijing, China). Equal amounts of protein samples were separated by sodium dodecyl sulfate-polyacrylamide gel electrophoresis (SDS-PAGE) and transferred to polyvinylidene fluoride membranes. Then, the membranes were blocked by 5% skim milk Tris-buffered saline containing 0.1% Tween (TBST) buffer for 90 min and washed with TBST buffer for 5 min. The membranes were incubated with the following primary antibodies: anti-GABA_A_R α1 (1:1,000, Abcam, Cambridge, UK), anti-BiP (1:1,000; Abcam), anti-pan-cadherin (1:2,000, Sigma, St. Louis, MO, USA), and anti-β-actin (1:10,000, Proteintech, Wuhan, China) overnight at 4°C. After washing with TBST 5 times (each for 5 min), the membranes were incubated with a secondary polyclonal antibody conjugated to horseradish peroxidase, anti-rabbit immunoglobulin G (IgG) (1:5,000, KPL, Gaithersburg, MD), and anti-mouse IgG (1:5,000, KPL) at room temperature for 1 h. The membranes were again washed 5 times (each for 5 min) and treated with an enhanced chemiluminescence detection kit (EMD Millipore, Billerica, MA, USA). The intensity of each band was quantified by densitometry using a gel image analysis software (Image Pro Plus, Media Cybernetics, USA). Relative expression was normalized to the expression of anti-pan-cadherin (1:2,000, Sigma) and anti-β-actin (1:10,000, Proteintech).

### Statistical Analysis

The data were analyzed using SPSS 20.0 software (IBM Corp., Armonk, NY, USA). Data are presented as the mean ± standard deviation (SD). Behavioral data were tested using a two-way analysis of variance (ANOVA) with repeated measures. Other data were analyzed using a one-way ANOVA with Tukey *post-hoc* comparisons. *P* < 0.05 was the criterion for statistical significance.

## Results

### Combination Treatment With 1% Isoflurane and 20 mg·kg^−1^·h^−1^ Propofol Protected Cognitive Function in Aging Rats With CH and Being Subjected to an ORIF Surgery

**Figure 1:**
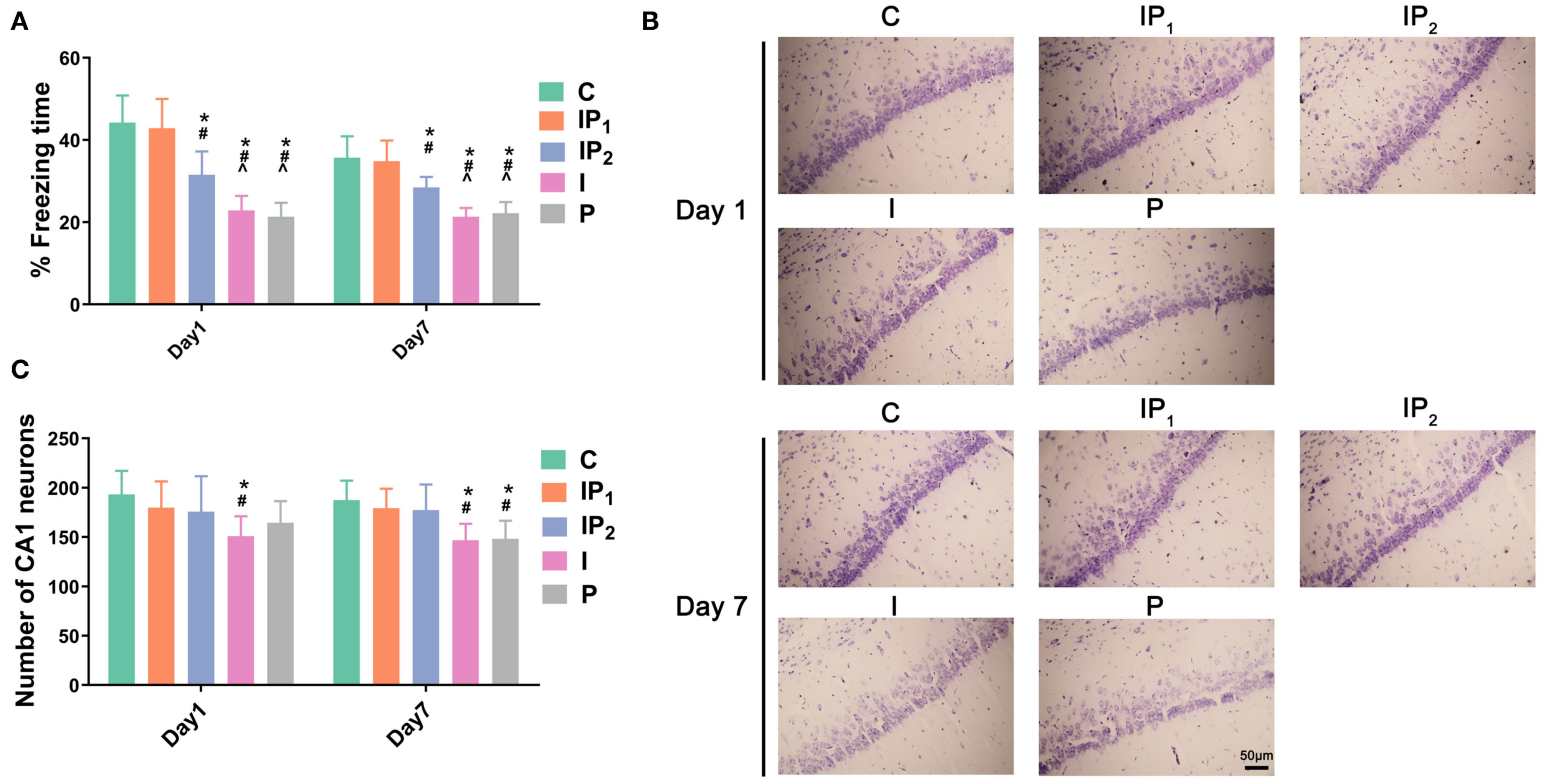
Combined treatment with 1% isoflurane and 20 mg·kg^−1^·h^−1^ propofol protected cognitive function and survival neurons in CH rats. **(A)** Hippocampus-dependent memory was evaluated as the percentage of freezing time on day 1 and day 7 after ORIF. Data are expressed as the mean ± SD (*n* = 8/group). Note that ORIF resulted in a significant reduction in the time of freezing behavior in the CA1 in Groups IP_2_, I, and P, which was prevented by the anesthetic schedule in Group IP_1_. **(B)** Nissl staining images of the hippocampal CA1 region were used to evaluate neuronal damage on day 1 and day 7 after ORIF. Note that ORIF resulted in a significant reduction in the number of remaining pyramidal neurons in the CA1 in Groups IP_2_, I, and P, which was prevented by the anesthetic schedule in Group IP_1_. **(C)** Quantification of surviving neurons in the CA1 on day 1 and day 7 after ORIF. Data are expressed as the mean ± SD (*n* = 8/group). *P < 0.05 compared with Group C; ^#^*P* < 0.05 compared with Group IP_1_; ^∧^*P* < 0.05 compared with Group IP_2_. Scale bars = 50 μm.

To observe the effects of different dosages of isoflurane and propofol on cognitive function, a contextual FC test was performed on the first and seventh days after ORIF. The percentage of freezing time in Group C and Group IP_1_ was not significantly different on the first day (C vs. IP_1_: 44.23 ± 6.60 vs. 42.86 ± 7.12, *P* = 1.00) or the seventh day (C vs. IP_1_: 35.70 ± 5.21 vs. 34.85 ± 5.02, *P* = 1.000) after ORIF (Figure 1A). However, in Groups IP_2_, I, and P, the percentage of freezing time was significantly reduced compared with Group C on day 1 (C vs. IP_2_: 44.23 ± 6.60 vs. 31.55 ± 5.68; C vs. I: 44.23 ± 6.60 vs. 22.86 ± 3.53; C vs. I: 44.23 ± 6.60 vs. 21.32 ± 3.42; all *P* < 0.05) and day 7 (C vs. IP_2_: 35.70 ± 5.21 vs. 28.48 ± 2.54; C vs. I: 35.70 ± 5.21 vs. 21.34 ± 2.12; C vs. I: 35.70 ± 5.21 vs. 22.16 ± 2.74; all *P* < 0.05) (Figure 1A). The results suggest that the combination of 1% isoflurane and 20 mg·kg^−1^·h^−1^ propofol could protect cognitive function, while other dosages could not.

### Treatments With Isoflurane or Propofol Alone Were Not Able to Prevent CA1 Neuronal Death in Aging Rats With CH That Were Subjected to ORIF Surgery

Hippocampal slices were stained with cresyl violet (Nissl staining) to investigate potential neuronal damage caused by anesthetics on days 1 and 7 after ORIF. Compared with Group C, the number of surviving neurons decreased 1 day after ORIF only in Group I (C vs. I: 193.13 ± 23.94 vs. 150.88 ± 20.19, *P* = 0.039, Figures 1B,C). On the seventh day after ORIF, the number of surviving neurons in Groups I and P was significantly lower than that in Group C (C vs. I: 187.38 ± 19.86 vs. 146.75 ± 16.70, *P* = 0.008; C vs. P: 187.38 ± 19.86 vs. 148.13 ± 18.39, *P* = 0.011). No significant changes were found in Groups IP_1_ and IP_2_ on day 1 (C vs. IP_1_: 193.13 ± 23.94 vs. 179.75 ± 26.60, *P* = 0.923; C vs. IP_2_: 193.13 ± 23.94 vs. 175.75 ± 35.94, *P* = 0.799) or day 7 (C vs. IP_1_: 187.38 ± 19.86 vs. 179.13 ± 19.96, *P* = 0.975; C vs. IP_2_: 187.38 ± 19.86 vs. 177.25 ± 26.02, *P* = 0.940) (Figures 1B,C).

### Combination Treatment With 1% Isoflurane and 20 mg·kg^−1^·h^−1^ Propofol Maintained the Expression Level of Cell GABA_A_R α1 in the Hippocampus

**Figure 2:**
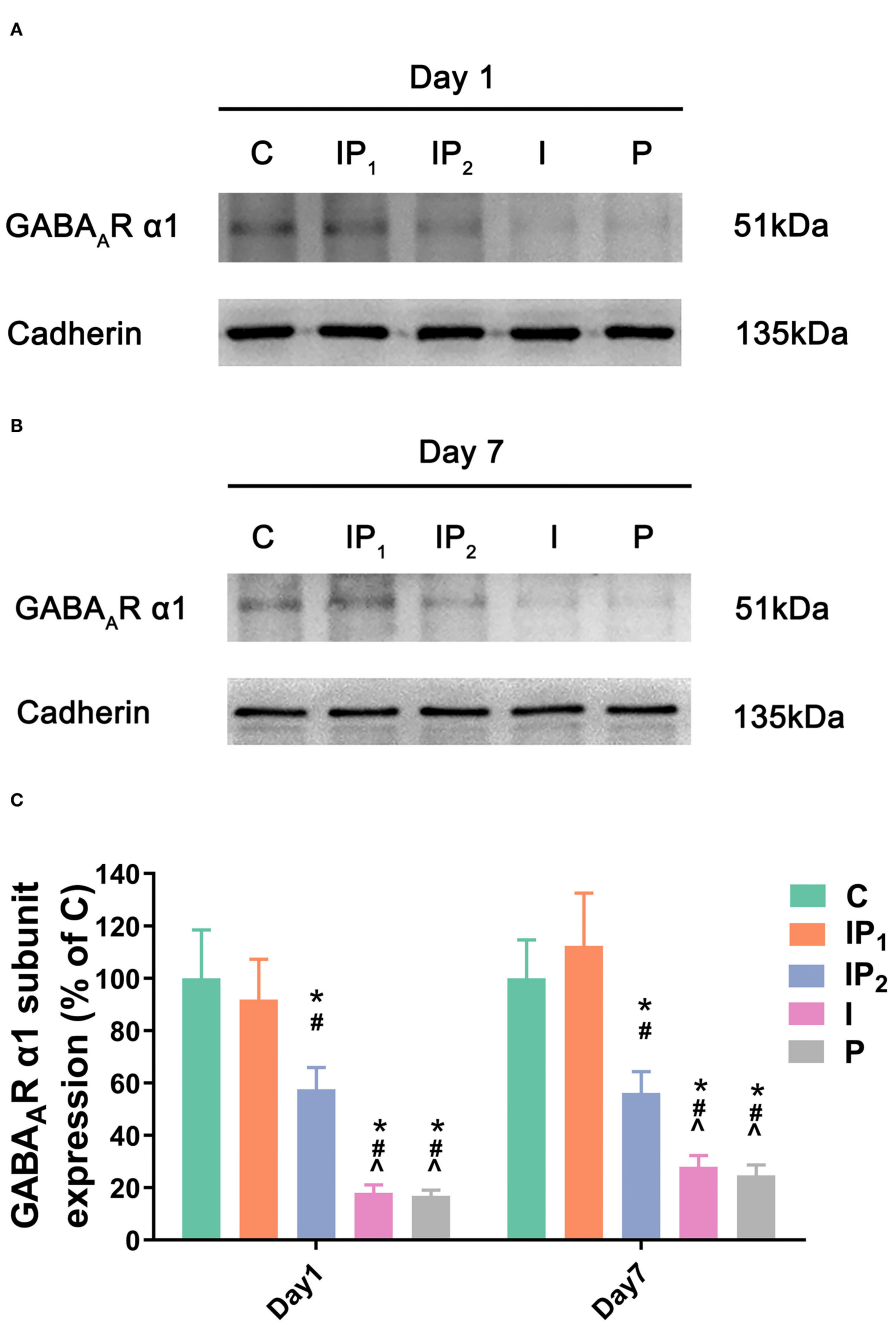
Combined treatment with 1% isoflurane and 20 mg·kg^−1^·h^−1^ propofol maintained the expression of the GABA_A_R α1 subunit. **(A,B)** The expression of the GABA_A_R α1 subunit in the hippocampus was determined by western blotting on day 1 and day 7 after ORIF. **(C)** Statistical graph of the expression of the GABA_A_R α1 subunit on day 1 and day 7 after ORIF. Data are expressed as the mean ± SD (*n* = 8/group). Note that ORIF resulted in a significant reduction in the expression of the GABA_A_R α1 subunit in the CA1 in Groups IP_2_, I, and P, which was prevented by the anesthetic schedule in the Group IP_1_. *P < 0.05 compared with Group C; ^#^*P* < 0.05 compared with Group IP_1_; ^∧^*P* < 0.05 compared with Group IP_2_.

As described above, GABA_A_R α1 is a key functional component of the neural substrate involved in cognitive functions. Therefore, western blotting was performed on the first and seventh days after ORIF to evaluate the membrane expression of the GABA_A_R α1 subunit. Cadherin was used as positive control membrane marker (42, 43). There was no difference in the expression of GABA_A_R α1 between Group C and Group IP_1_ on day 1 (C vs. IP_1_: 100.00 ± 18.48 vs. 91.86 ± 15.45, *P* = 0.629) or day 7 (C vs. IP_1_: 100.00 ± 14.72 vs. 112.39 ± 20.17, *P* = 0.261) after ORIF. The expression of GABA_A_R α1 was downregulated after ORIF in Groups IP_2_, I, and P compared with Group C on day 1 (C vs. IP_2_: 100.00 ± 18.48 vs. 57.57 ± 8.39, *P* < 0.005; C vs. I: 100.00 ± 18.48 vs. 18.02± 3.07, *P* < 0.001; C vs. P: 100.00 ± 18.48 vs. 16.90 ±3.45, *P* < 0.001) and day 7 (C vs. IP_2_: 100.00 ± 14.72 vs. 56.23 ± 8.12, *P* < 0.001; C vs. I: 100.00 ± 14.72 vs. 27.92 ± 4.39, *P* < 0.001; C vs. P: 100.00 ± 14.72 vs. 24.71 ±4.01, *P* < 0.001) (Figure 2).

### Combination Treatment With 1% Isoflurane and 20 mg·kg^−1^·h^−1^ Propofol Protected Neurons From ER Stress-Related Damage

**Figure 3:**
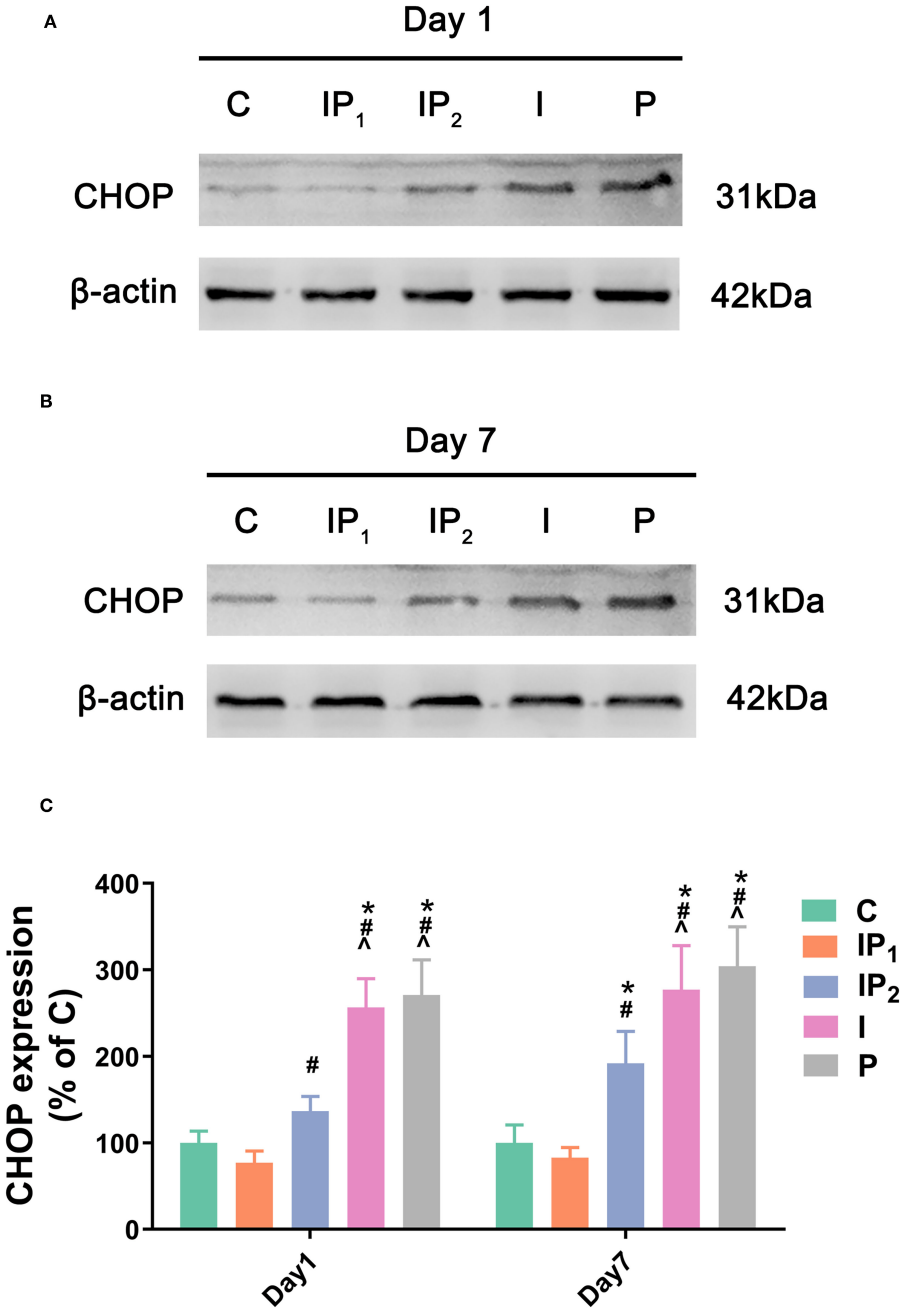
Combined treatment with 1% isoflurane and 20 mg·kg^−1^·h^−1^ propofol prevented ER stress-related damage. **(A,B)** The expression of CHOP in the hippocampus was determined by western blotting on day 1 and day 7 after ORIF. **(C)** Statistical graph of the expression of CHOP on day 1 and day 7 after ORIF. Data are expressed as the mean ± SD (*n* = 8/group). Note that ORIF resulted in a significant increase in the expression of CHOP in the CA1 in Groups IP_2_, I, and P, which was prevented by the anesthetic schedule in Group IP_1._ *P < 0.05 compared with Group C; ^#^*P* < 0.05 compared with Group IP_1_; ^∧^*P* < 0.05 compared with Group IP_2_.

To analyse ER stress-related damage, the expression of CHOP was evaluated by western blotting. There was no difference between Group C and Group IP_1_ on day 1 (C vs. IP_1_: 100.00 ± 13.63 vs. 76.93 ± 13.74, *P* = 0.409) or day 7 (C vs. IP_1_: 100.00 ± 20.70 vs. 82.77 ± 11.96, *P* = 0.876). Compared with Group C, the expression of CHOP in Group IP_2_ did not markedly change on the first day (C vs. IP_2_: 100.00 ± 13.63 vs. 136.70 ± 17.07, *P* = 0.058) but increased markedly on the seventh day after ORIF (C vs. IP_2_: 100.00 ± 20.70 vs. 191.85 ± 37.16, *P* < 0.001). The expression of CHOP was significantly upregulated in Groups I and P on day 1 (C vs. I: 100.00 ± 13.63 vs. 256.72 ± 33.15, *P* < 0.001; C vs. P: 100.00 ± 13.63 vs. 270.81 ± 40.61, *P* < 0.001) and day 7 (C vs. I: 100.00 ± 20.70 vs. 277.16 ± 50.77, *P* < 0.001; C vs. P: 100.00 ± 20.70 vs. 304.08 ± 45.71, *P* < 0.001) after ORIF (Figure 3).

### 1% Isoflurane and 20 mg·kg^−1^·h^−1^ Propofol Protect Neurons by Elevating the Expression of BiP

**Figure 4:**
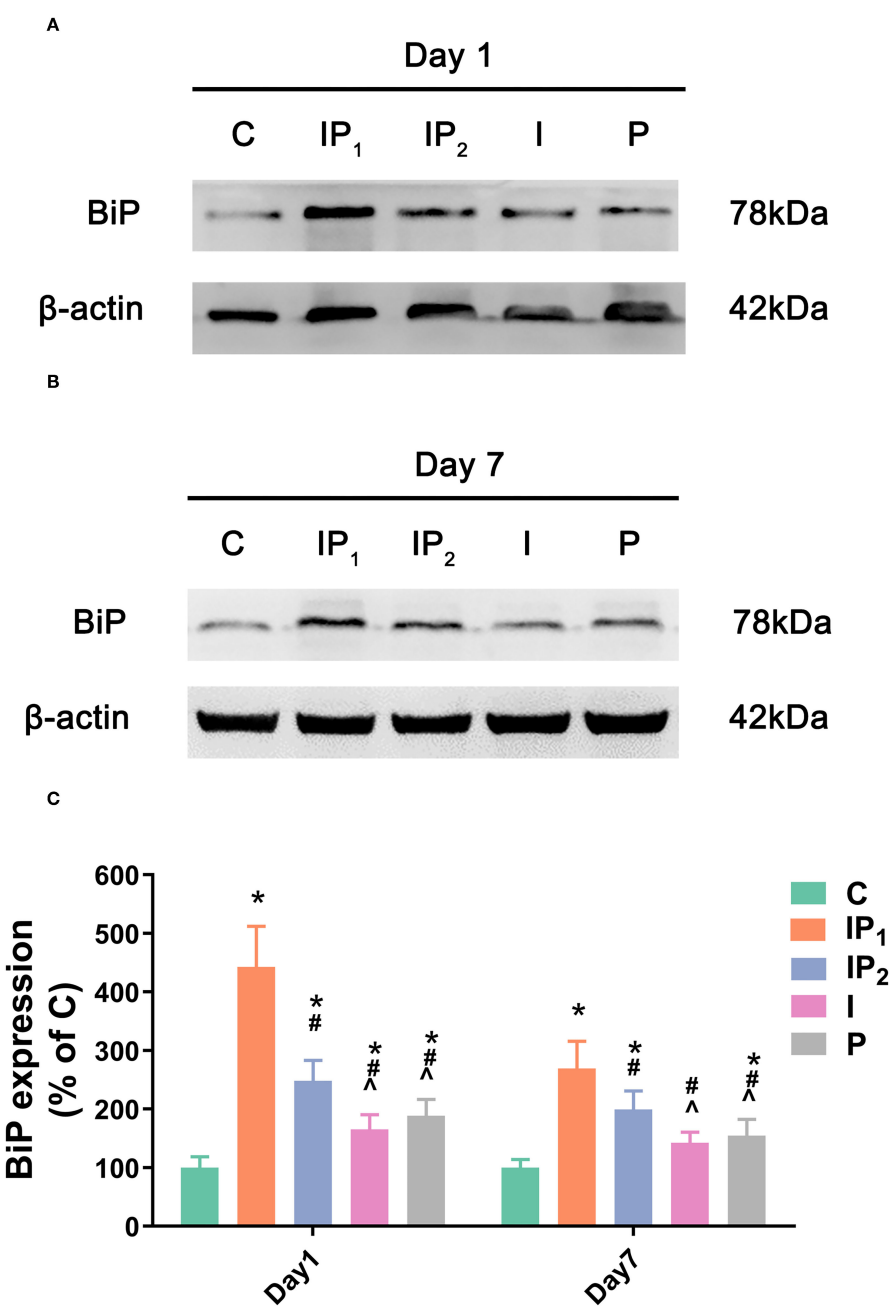
Combined treatment with 1% isoflurane and 20 mg·kg^−1^·h^−1^ propofol maintained the adaptive ability of neurons by increasing the expression of BiP. **(A,B)** The expression of BiP in the hippocampus was determined by western blotting on day 1 and day 7 after ORIF. **(C)** Statistical graph of the expression of BiP on day 1 and day 7 after ORIF. Data are expressed as the mean ± SD (*n* = 8/group). Note that ORIF resulted in a significant increase in the expression of BiP in the CA1 in Groups IP_2_, I, and P, which was prevented by the anesthetic schedule in Group IP_1_. *P < 0.05 compared with Group C; ^#^*P* < 0.05 compared with Group IP_1_; ^∧^*P* < 0.05 compared with Group IP_2_.

The expression levels of BiP in Groups IP_1_, IP_2_, I, and P were all upregulated compared with that in Group C on day 1 (C vs. IP_1_: 100.00 ± 18.58 vs. 442.86 ± 69.09, C vs. IP_2_: 100.00 ± 18.58 vs. 248.02 ± 35.15, C vs. I: 100.00 ± 18.58 vs. 165.13 ± 25.53, C vs. P: 100.00 ± 18.58 vs. 188.54 ± 27.90, all *P* < 0.05). The highest expression level was found in Group IP_1_, and the lowest expression level was found in Group I. On day 7, the expression of BiP fell in all four groups, and there was no significant difference between Group I and Group C (C vs. I: 100.00 ± 13.91 vs. 142.57 ±18.70, *P* = 0.053). However, the expression of BiP in Groups IP_1_, IP_2_, and P was significantly higher than that in Group C (C vs. IP_1_: 100.00 ± 13.91 vs. 268.27 ± 46.51, C vs. IP_2_: 100.00 ± 13.91 vs. 199.47 ±31.66, C vs. P: 100.00 ± 13.91 vs. 154.64 ± 27.93, all *P* < 0.05, Figure 4). The highest expression was observed in Group IP_1_ (Figure 4).

## Discussion

In our study, aging (16–18 month) rats were chosen as test subjects. All rats received ligation of the bilateral CCA to mimic the pathological process of CAS. Thirty days after ligation surgery, ORIF surgery was performed under different anesthesia regimes according to the group. After ORIF surgery, behavioral experiments (FC test) were carried out to evaluate the cognitive function of the rats. Histological analyses (Nissl staining) were performed to explore neuronal damage, and biochemical analyses (western blotting) of harvested rat brain tissues were performed to detect molecular changes.

The first consideration that must discussed is the selection and intervention of the test subject. The incidence of PND in orthopedic patients varies from 16 to 45%, although it can be as high as 72% (44), and it has been proven that aging is a risk factor (45). Therefore, we chose aging rats as test subjects. CH has been reported to be a key factor in the development of cognitive impairment (7). The underlying mechanism could be hypoxia-induced white matter damage, microvascular inflammation, and neuro-glio-vascular dysfunction (6). We deem that aging patients with perioperative CH require more attention to be paid to the selection of surgery and anesthesia. Moreover, CAS has been detected in 75% of men and 62% of women older than 65 years, with a prevalence of ≥50% stenosis of 7% in men and 5% in women (46). Taking incidence into account, we therefore used ligation of the CCA to induce CH in aging rats in this study. Moreover, our previous study has confirmed that ligation of the CCA contributes to cognitive impairment and histopathologic changes in aging rats (47). As it is difficult to separate clinical anesthesia and surgery, and our main purpose was to explore the combined effects of the two factors, no separate anesthesia group was used, which is consistent with most recent studies (48–50).

The FC test is a very sensitive and effort-independent test of learning and memory (51). To eliminate effects on motor ability caused by tibial fracture, the FC test was chosen to inspect cognitive function after ORIF surgery. Isoflurane has been reported to suppress learning in a dose-dependent fashion. Hence, we trained animals before surgery and anesthesia to remove the influence of the acquisition phase on the assessment of memory postoperatively (36). After ORIF surgery and anesthesia, the rats were placed in the same chamber that was used during the FC training phase. No tone or shock was delivered while the rats were in the chamber. In this circumstance, freezing behaviors rely on hippocampal memory (35, 37). It was demonstrated that medial temporal lobe regions, including the hippocampus, are most commonly affected in mild cognitive impairment and early AD (52–54). Thus, in our study, we focus on measurement of hippocampus-dependent memory. We found that the freezing time of rats was significantly shorter in Groups I, P, and IP_2_ than in Group C, while there was no obvious difference between Groups C and IP_1_. The only difference in the intervention among Groups I, P, IP_2_, and IP_1_ was the anesthesia method. Our results suggest that hippocampal-dependent memory impairment was not exhibited by the rats anesthetized with 1% isoflurane and 20 mg·kg^−1^·h^−1^ propofol, in contrast to all other groups of rats. Such an obvious difference aroused our interest in evaluating the state of related anatomic structures.

The hippocampal CA1 area is crucial for context-specific memory retrieval and spatial memory. After CA1 lesions, both recent and remote memory are impaired (55). Furthermore, this area is vulnerable to ischaemia injury (56). Thus, we chose the hippocampal CA1 area to measure the number of survival neurons and the expression of certain protein. On day 1, the number of neurons in Group I decreased obviously compared with Group C, and on day 7, the number of neurons in Groups I and P were markedly decreased compared with Group C. The difference between Group C and the combined anesthesia groups was not significant. Thus, we can draw the conclusion that, compared with the combination groups, the high dose of isoflurane or propofol alone can cause irreversible damage to the nervous system.

The GABA_A_R α1 subunit has also been linked to brain cognitive functions (57). More recently, the expression level of GABA_A_R α1 in the hippocampal CA1 region was found to be significantly downregulated in rats with chronic ischaemic encephalopathy (57). Proteostasis of GABA_A_R α1 subunit highly relies on ER function. Neuronal failure of the proteostasis network may cause protein aggregation that leads to neurodegeneration (58, 59). In our study, the expression of GABA_A_R containing the α1 subunit decreased in all but one group (the 1% isoflurane and 20 mg·kg^−1^·h^−1^ propofol group). It indicated that protein homeostasis was altered in all but not IP_1_ groups, which was coincident with the change in freezing time.

Accumulated evidence could support our study. Previous *in vitro* study showed that high dose isoflurane (treatment at a dose of 2% for 6 h) induced apoptosis by causing ER stress through ryanodine receptors but lower dose isoflurane (treatment at a dose of 1% for 1, 3, 6 h) did not (60). *In vivo* study suggested that ER stress-mediated apoptotic pathway was involved in isoflurane (treatment at a dose of 1.3% for 4 h) neurotoxicity in aged rats. Inhibition of ER stress overactivation contributed to the relief of isoflurane-induced histopathologic changes (61). Moreover, Coghlan et al. confirmed that the induction of ER stress by isoflurane (treatment at a dose of 1.1% for 4 h) occurred after the initiation of protein misfolding (62). These results indicate that cytotoxicity of isoflurane is in a dose-dependent way and related to ER stress. Analogously, propofol has a dose-dependent neuroprotective effect. Our previous study showed that propofol at doses of 10 or 20 mg·kg^−1^·h^−1^ infused at the onset of reperfusion for 30 min could provide neuroprotection to transient MCAO rats but 30 mg·kg^−1^·h^−1^ could not (12). Another study showed that infusion of propofol (36 or 72 mg·kg^−1^·h^−1^) resulted in aggravation of neurologic dysfunction, increased 28-day mortality rate, and impaired posttraumatic neurogenesis (63). *In vitro* study showed that the neuroprotective effect of propofol increased in a dose-dependent manner within 10 uM and decreased in a dose-dependent manner beyond 10 uM. Increase of endogenous BiP was the key of propofol's neuroprotection (64). In the present study, we only quantified CHOP and BiP. However, it is these two key factors that could confirm the neuroprotection of 1% isoflurane and 20 mg·kg^−1^·h^−1^ propofol. BiP, the key molecular chaperone in the ER, can help to alleviate ER stress and maintain calcium homeostasis (65), overexpression or induction of BiP possesses anti-apoptosis potential (66, 67). In our experiments, the expression of BiP was the highest in rats anesthetized with 1% isoflurane and 20 mg·kg^−1^·h^−1^ propofol among all four general anesthesia groups. CHOP is acknowledged as a specific transcription factor of ER stress (27). Unlike ER chaperones, CHOP is not generally synthesized under normal physiological conditions, or is present in the cytosol at very low levels under non-stressed conditions. Stress leads to the induction of CHOP and its accumulation in the nucleus (68). CHOP overactivation was closely related to neurodegenerative disease (28). In consistent with this, our study showed that elevated expression of CHOP in all but not IP_1_ groups. In group I and P, expression of CHOP was extremely high, while the mount of surviving neurons showed a distinguished decrease, which could provide a more intuitionistic result of cell damage.

In this study, hippocampus-dependent memory of rats in group IP_1_ was not impaired, and expression level of GABA_A_R α1, a key cognition-related protein, remained normal. ER stress alleviator, BiP, increased extremely while ER stress transcription factor, CHOP, showed no statistical difference compared with the control group. Numbers of surviving neurons confirmed the substantial neuronal damage caused by propofol or isoflurane alone.

Taking the above results into consideration, we consider that 1% isoflurane and 20 mg·kg^−1^·h^−1^ propofol is a more favorable aesthetic combination to avoid further damage to cognitive function of aging rats with CH during orthopedic surgery. The potential mechanism of this phenomenon may be related to alleviation of ER stress, but it remains to be verified.

With the advent of the aging society, clinical anesthesia is facing a variety of complex challenges, more exploration is needed to ensure the overall safety of patients. The harm caused by the application of large dose of a single anesthetic drug has been paid more and more attention. Therefore, this experiment explores the combined application of low-dose anesthetics to ensure the safety of anesthesia while minimizing the adverse effects of drugs, so as to provide new ideas for the practical clinical work. The experimental results show that the combination of low-dose anesthetics can enhance the protection of cognitive function. At the same time, more comprehensive and in-depth research is needed to explore related mechanisms and lay a solid foundation for personalized and precise anesthesia.

## Data Availability Statement

The raw data supporting the conclusions of this article will be made available by the authors, without undue reservation.

## Ethics Statement

The animal study was reviewed and approved by Institutional Animal Care and Use Committee of Tianjin Medical University.

## Author Contributions

HW helped with conception and design, acquisition, analysis and interpretation of the data, critical revision of the article, and giving final approval. XB and TL helped with conception and design, acquisition, analysis and interpretation of the data, drafting and critical revision of the article, and giving final approval. ZX, ZY, and GW helped with critical revision of the article and giving final approval. DG helped with analysis and interpretation of data, acquisition, critical revision of the article, and giving final approval. JW, YS, and CY helped with conception and design, analysis and interpretation of the data, critical revision of the article, and giving final approval. GL and JM helped with analysis and interpretation of the data, critical revision of the article, and giving final approval. All authors contributed to the article and approved the submitted version.

## Conflict of Interest

The authors declare that the research was conducted in the absence of any commercial or financial relationships that could be construed as a potential conflict of interest.

## Abbreviations

BiP: binding immunoglobulin protein
CCA: common carotid arteries
CH: cerebral hypoperfusion
CHOP: C/EBP homologous protein
CNS: central nervous system
ER: endoplasmic reticulum
ERAD: endoplasmic reticulum-associated degradation
FC: fear conditioning
GABA: γ-aminobutyric acid
GABA_A_R: γ-aminobutyric acid A type receptor
MAC: minimum alveolar concentration
mIPSC: miniature inhibitory postsynaptic current
OGD: oxygen-glucose deprivation
ORIF: open reduction and internal fixation
PND: perioperative neurocognitive disorders
ROS: reactive oxygen species
UPR: unfolded protein response.
